# A pharmacological approach to investigating effector translocation in rice-*Magnaporthe* oryzae interactions

**DOI:** 10.1080/15592324.2024.2350869

**Published:** 2024-05-09

**Authors:** Ely Oliveira-Garcia, Allison Jane Hamilton

**Affiliations:** Department of Plant Pathology and Crop Physiology, Louisiana State University Agricultural Center, Baton Rouge, LA, USA

**Keywords:** Golgi-independent secretion, clathrin-mediated endocytosis (CME), clathrin heavy chain, endosidin9 (ES9), endosidin9-17 (ES9-17), effector translocation, biotrophic interfacial complex (BIC), *Magnaporthe oryzae* (syn. *Pyricularia oryzae*)

## Abstract

Fungal pathogens deliver effector proteins into living plant cells to suppress plant immunity and control plant processes that are needed for infection. During plant infection, the devastating rice blast fungus, *Magnaporthe oryzae*, forms the specialized biotrophic interfacial complex (BIC), which is essential for effector translocation. Cytoplasmic effectors are first focally secreted into BICs, and subsequently packaged into dynamic membranous effector compartments (MECs), then translocated via clathrin-mediated endocytosis (CME) into the host cytoplasm. This study demonstrates that clathrin-heavy chain inhibitors endosidin-9 (ES9) and endosidin-9–17 (ES9–17) blocked the internalization of the fluorescently labeled effectors Bas1 and Pwl2 in rice cells, leading to swollen BICs lacking MECs. In contrast, ES9–17 treatment had no impact on the localization pattern of the apoplastic effector Bas4. This study provides further evidence that cytoplasmic effector translocation occurs by CME in BICs, suggesting a potential role for *M. oryzae* effectors in co-opting plant endocytosis.

## Introduction

The mechanism by which cytoplasmic effectors from eukaryotic filamentous pathogens (fungi and oomycetes) are translocated across the plant plasma membrane to control living plant cells has remained elusive for over two decades.^1–5^ The rice-*Magnaporthe oryzae* pathosystem is a particularly good model to address this fundamental question of molecular-plant microbe interactions. The rice-*Magnaporthe oryzae* pathosystem’s effectors can be tracked inside rice cells using live cell imaging of fluorescently labeled effector proteins, allowing effector translocation to be better understood.^5–7^ *M. oryzae* is the causal agent of blast diseases of rice, wheat, and other cereal crops and grasses.^8^ *M. oryzae* executes a hemibiotrophic lifestyle in which intracellular invasive hyphae undergo successive colonization of living rice cells.^9^ Unlike that of oomycete effectors, blast cytoplasmic effectors are not reported to contain a translocation motif.^5^ Instead, the major predictor of translocation into the rice cell is accumulation of effectors in the Biotrophic Interfacial Complex (BIC), a specialized interfacial structure.^2,5,10^ Biotrophic invasive hyphae growing in living host cells are surrounded by an extra-invasive hyphal membrane (EIHM), which forms an enclosed EIHM compartment. Apoplastic effectors, such as biotrophy-associated secreted protein 4 (Bas4), LysM protein 1 (Slp1), and Bas113, are secreted through the conventional Golgi-dependent secretion pathway (Brefeldin A sensitive). Apoplastic effectors accumulate throughout the EIHM matrix, outlining the entire invasive hypha.^2,5–7,11^ In contrast, cytoplasmic effectors become concentrated in BICs, the specialized interfacial region surrounded by rice cytoplasm.^7,10,12^ The cytoplasmic effectors include known AVR effectors, such as Pwl2, Avr-Pita, and AvrPiz-t, as well as many other effectors that trigger susceptibility in plants.^10,13^ Using a specialized, Golgi-independent secretion system, the BIC-associated cells, primary hypha, and first bulbous invasive hyphal cell focally secrete cytoplasmic effectors into BICs using a specialized, Golgi-independent secretion system.^2,14^ Targeted gene replacement mutants showed that the *M. oryzae* exocyst components E×o70and Sec5 were required for efficient secretion of effectors into BICs, although secretion of apoplastic effectors appeared normal.^14^ In *Δexo70* and *Δsec5* mutants, BIC-localized effectors were partially retained inside the BIC-associated invasive hypha cells. Fluorescence Recovery After Photobleaching (FRAP) experiments and localization of protein secretion machinery components in BIC-associated cells showed that secretion of effectors into BICs continues while invasive hyphae grow to colonize the rice cells.^2,10^

The accumulation patterns of cytoplasmic effectors in BICs indicated that BICs are involved in effector translocation. High resolution images of fluorescently labeled effectors in BICs revealed that cytoplasmic effectors are packed in vesicle-like structures. Since many of these structures can reach sizes larger than 100 nm, they were named Membranous Effector Compartments (MECs).^6^ The localization pattern of effectors in MECs indicates that cytoplasmic effectors are internalized via endocytosis. For endocytosis to occur in BICs, the BIC must have large amounts of plant membrane and clathrin. Indeed, localization assays using transgenic rice expressing Lit6b (LOW-TEMPERATURE INDUCED PROTEIN 6B; plant plasma membrane marker) fused to green fluorescent protein (LTi6B:GFP) reveals that BICs are plant plasma membrane enriched structures. Remarkably, LTi6B:GFP clearly outlines the individual MECs.^6^ Moreover, localizations assays with transgenic rice lines expressing CLC1 (CLATHRIN LIGHT CHAIN1):eGFP (Clathrin-mediated endocytosis marker) also showed that BICs are plant plasma clathrin-rich structures. Importantly, colocalization assays between fluorescently labeled effectors and CLC1 showed that MECs colocalizes with plant clathrin in BICs, confirming the hypothesis that CME focally occurs in BICs.^6^ Further confirmation of the impact of CME can be obtained using endocytosis inhibitors in rice cells undergoing infection by *M. oryzae* expressing fluorescently labeled effectors. Here, we employed two major CLATHRIN HEAVY CHAIN inhibitors, ES9 and ES9–17 to dissect the impact of CME on the translocation of cytoplasmic effectors of *M. oryzae* in plant cells.

## Materials and methods

### Fungal strains, fungal transformation, and live cell imaging of M. oryzae effectors in planta

Using an *Agrobacterium tumefaciens*-mediated transformation protocol described previously,^15^ strain FLG149 was generated by transformation of Kv91^12^ with pBV440.^12^ FLG149 and KV217^6^ were primarily used to analyze ES9 and ES9–17 CME inhibitor assays.

Rice leaf sheath inoculations were performed as described^6,7^ using the susceptible rice variety YT16. Each segment was trimmed at 29 hours post inoculation (hpi), treated for 3 hours with specific inhibitors, or imaged immediately by laser confocal microscopy. Biological replicates were performed as independent experiments using fungal cultures fresh out of frozen storage and new rice plants. All conclusions are supported by at least 3 biological replicates, with each replication including observation of ∼100 infection sites.

Confocal imaging was performed with a Leica SP8 confocal microscope system with a water immersion objective (HC PL APO 63×/1.20 WCorr). Excitation/emission wavelengths were 500 nm/505 to 540 nm for eYFP and 543 nm/560 to 615 nm for mRFP. Image acquisition and processing, and fluorescence intensity line scans were generated using Leica LAS X software to analyze ES9 and ES9–17 CME inhibitor assays.

### Treatment with pharmacological inhibitors

To examine the effects of endocytosis inhibitors on *in planta* effector uptake, inoculated, trimmed rice sheaths were incubated at 25°C with endosidin9 (ES9) (10 µM)^16^ and endosidin9–17 (ES9–17) (30 µM),^17^ (all from Sigma) solution for 1 to 5 hours. Negative controls were performed with 0.1% DMSO.

### Statistical analysis

All experiments were performed with at least 3 biological replications, which were independent experiments with fungal cultures directly grown from frozen storage and different rice plantings. Biological replications included at least 2 technical repeats (independent assays with the same biological materials) to further confirm the reproducibility of the data. In all cases, technical and biological replications gave consistent results. Sample sizes, number of biological replicates, and the statistical tests used in each experiment are specified in the figure legends. Data were analyzed using an unpaired 2-tailed Student’s t-test. *p* = 0.05 was considered nonsignificant, and exact values are shown where appropriate. All statistical analysis was performed using R Statistical Software (version 4.1.2) and Prism10 (GraphPad). Bar graphs show the mean ± s.e.m. (unless otherwise stated) and were generated using Prism10 (GraphPad).

## Results and discussion

**Figure 1.**
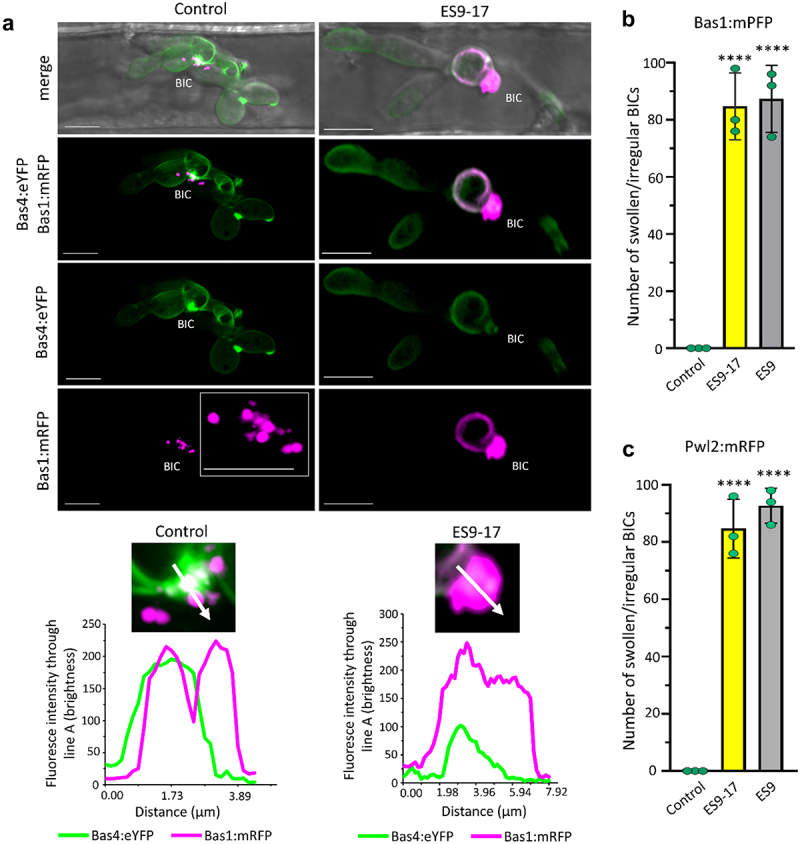
Treatment with CME inhibitors impacts localization of the cytoplasmic effector, but not the apoplastic effector in mature BICs at 32 hpi. A. each panel displays side-BICs formed during rice leaf sheath cell invasion by strain FLG149 expressing Bas1:mRFP and Bas4:eYFP. Relative localizations are shown by fluorescence intensity linescans for YFP (green) and mRFP (magenta) along the path of the white arrow. Inhibitor treatments were initiated at 32 hpi. In the untreated control, apoplastic effector Bas4:eYFP identifies an inner base layer in the BIC relative to the outer MEC structure for cytoplasmic effector Bas1:mRFP. One MEC appears separated from the BIC (magenta puncta). Treatment with CLATHRIN HEAVY CHAIN inhibitor ES9–17 results in swollen, irregular localization of Pwl2:mRFP in BICs lacking obvious MEC puncta. Bas4:eYFP localization appears unaffected. From top to bottom, images show merged bright field, eYFP and mRFP fluorescence; merged eYFP and mRFP; eYFP; mRFP fluorescence; insets with enlarged mRFP view; and intensity line scans along the paths of the white arrows in insets. Images are projections of confocal optical sections. Ap, appressorium; PH, primary hypha. Scale bars = 10 μm. B. quantification of swollen, irregular-shaped BICs formed by *M. oryzae* strain KV170 expressing Bas1:mRFP after treatment with ES9–17 and ES9. Inhibitor treatments were initiated at 32 hpi. ***p = 0.0004 (ES9), ***p = 0.0004 (ES9–17). C. quantification of swollen, irregular-shaped BICs formed by *M. oryzae* strain KV209 expressing Pwl2:mRFP after treatment with ES9–17 and ES9. Inhibitor treatments were initiated at 32 hpi. ***p = 0.0003 (ES9), ***p = 0.0002 (ES9–17).

**Figure 2.**
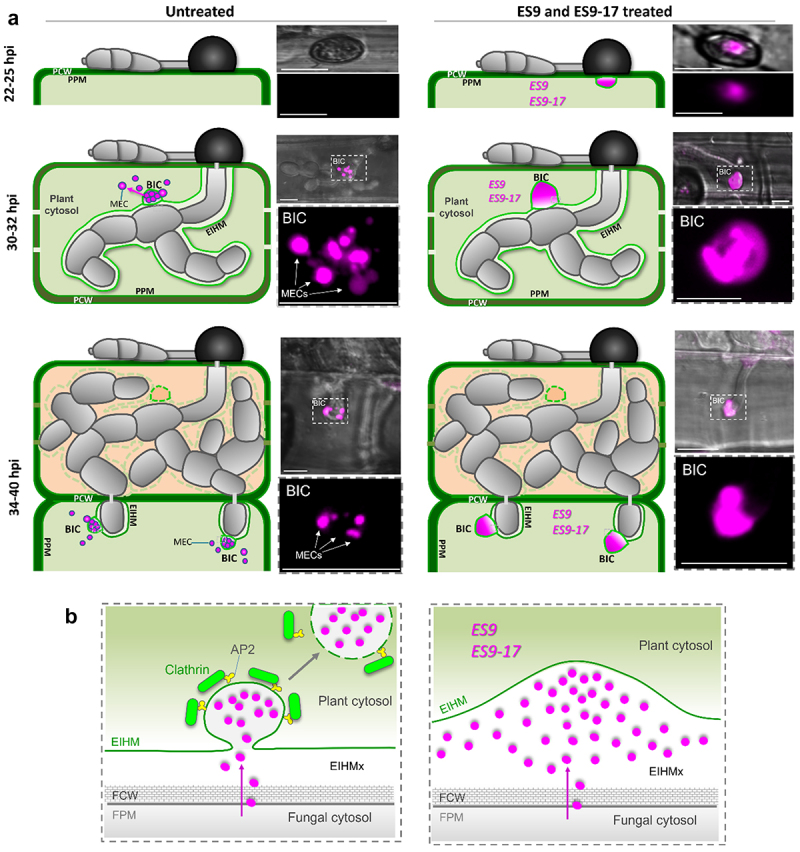
Endosidin-9 and endosidin-9–17 inhibit plant clathrin heavy chain function and block internalization of *M. oryzae* effectors in plant cells. A. model for cytoplasmic effector translocation via host CME based on blocking of BIC function through clathrin heavy chain inhibitors ES9 and ES9–17 associated with endocytosis. Upper panels represent a rice cell undergoing infection by *M. oryzae* at 22–25 hpi (upper cells), 30–32 hpi (middle cells), and 36–40 hpi (lower cells) under treatment or not with ES9 and ES9–17. Cytoplasmic effector-labeled MECs in BICs of strain KV170 expressing Bas1:mRFP (magenta) while invading a rice sheath epidermal cells under treatment of ES9–17, a more specific version of ES9. The lower panels (corresponding to the white box with dashed lines) show Bas1:mRFP fluorescence channels. Scale bars are 10 µm. Clathrin heavy chain inhibitors block effector translocation from early stages of appressorial penetration and in BICs from invaded cells. Visualization of MEC and CME inhibition is easier in early stages of infection (26–30 hpi) due to the large dimensions of BICS. Once the fungus crosses the neighboring cell, each invasive hyphae will care a smaller, but active BIC and MEC tend to be smaller than in the first invaded cell. B. the lower right panel represents clathrin mediated endocytosis of effectors at BICs. The lower left panel represents effector retention and effector translocation blockage at BICs under treatment of ES9 and ES9–17, thereby leading to the swollen or irregular BIC phenotype. A-B. BIC, biotrophic interfacial complex; MEC, membranous effector compartment; AP2, adaptor protein-2 complex; EIHMx, extra-invasive hyphal matrix; FPM, fungal plasma membrane; FCW, fungal cell wall. hpi, hours-post inoculation.

Pharmacological approaches have facilitated studies in diverse biological systems (Robinson et al. 2008; Hicks and Raikhel 2010; Grassart et al. 2014; Fan et al. 2015).^7,17^ Two pharmacal inhibit the function of CLATHRIN HEAVY CHAIN (CHC), Endosidin9 (ES9) and Endosidin9–17 (ES9–17).^17^ ES9 was reported to inhibit CHCs in Arabidopsis and Drosophila. However, ES9 also causes cytoplasmic acidification and has a proton translocator activity.^17^ ES9–17 was reported to inhibit CHCs in Arabidopsis and human cells.^17^ ES9–17, a more specific version of ES9, lacks cytoplasmic acidification activity.^17^ To date, no other clathrin heavy chain specific inhibitors have been reported. To assess the potential role of plant endocytic machinery in cytoplasmic effector translocation, we tested the impact of ES9 and ES9–17 on effector translocation in mature BICs (32 hours after inoculation). We used ES9 and ES9–17 concentrations previously described in Arabidopsis^17^ and a time of exposure of 3 hours, which shows optimal effects on BIC morphology (Figure 1). Compared to the untreated control (Figure 2a), treatment with the CME inhibitors ES9–17 and ES9, led to the formation of abnormally shaped, swollen BICs that lacked punctate MECs (Figure 1a). Fluorescence intensity line scans showed distances that were 2-fold greater for Pwl2:mRFP after ES9–17 treatment, with little (if any) impact on the localization of Bas4:eGFP in the base of the BIC (Figure 1a). Pwl2:mRFP and Bas1:mRFP localization patterns were quantified during the infection of rice treated with ES9–17 and ES9 (Figure 2b). Compared to the untreated controls, treatment with ES9–17 and ES9 inhibitors generated a high proportion of swollen, irregularly shaped BICs lacking punctate MECs (Figure 1b,c). We previously showed the ES9 and ES9–17 treatment leads to Pwl2:mRFP accumulates under appressorial pores, suggesting that effector translocation occurs at early stages of appressorial penetration (Figure 2a). As infection progresses, the rice blast fungus continues to deliver effectors via CME though a single BIC, fills the invaded cell and as the fungus crosses to subsequent neighboring cells using multiple BICs (Figure 2a).

Our data demonstrated that CME focally occurs in the BIC, and ES9–17 and ES9 inhibitors impair effector translocation (Figures 1 and 2). We also hypothesized that these clathrin heavy chain inhibitors block the formation of coated MEC, thereby blocking the translocation of effectors between BIC to rice cell cytoplasm (Figure 2b). Consistent with these findings, recent study on *Phytophthora infestans* effectors also demonstrated that CME is critical for the translocation of RXLR effectors into plant cells.^18,19^ The findings of the present study have confirmed that inhibition of rice CHATRIN HEAVY CHAINs impair BIC functionality. Our data collectively suggests that the blast fungus co-opts CME for the internalization of *M. oryzae* effectors inside living rice cells. Further confirmation will come through continuing research to determine how the endocytic machinery is recruited to BICs and how it interacts with effector cargos. The identification of effectors with critical roles in the effector internalization process, including effectors involved in disrupting MECs and releasing effectors into the rice cytoplasm is of high priority. It is important to understand how MECs are transported from BICs to rice cell cytoplasm. Finally, the fundamental knowledge of the molecular mechanisms of fungal effector translocation can be translated into novel strategies to control blast diseases in major cereal crops worldwide.

## Supplementary Material

Supplemental Material
