# Supplementary material for: A pharmacological approach to investigating effector translocation in rice-Magnaporthe oryzae interactions
Source: Plant Signal Behav. 2024 May 9;19(1):2350869. doi: 10.1080/15592324.2024.2350869 (PMC11085958; doi:10.1080/15592324.2024.2350869)
Supplement: Supplementary file 1 [file KPSB_A_2350869_SM4997.odt]

Supplemental Material

A graph of es9 and es9

Description automatically generated

Figure S1. Influence of time of exposure of ES9 and ES9-17 on BIC morphology and effector translocation. N=100 BICs
